# Amount of Information Needed for Model Choice in Approximate Bayesian Computation

**DOI:** 10.1371/journal.pone.0099581

**Published:** 2014-06-24

**Authors:** Michael Stocks, Mathieu Siol, Martin Lascoux, Stéphane De Mita

**Affiliations:** 1 Department of Ecology and Genetics, Program in Plant Ecology and Evolution, Evolutionary Biology Centre, Uppsala University, Uppsala, Sweden; 2 Institut National de la Recherche Agronomique (INRA), UMR Agroécologie, Dijon, France; 3 Institut National de la Recherche Agronomique (INRA), UMR Interactions Arbres-Microorganismes (IAM), Nancy, France; 4 Institut de Recherche pour le Développement (IRD), UMR Diversité et Adaptation des Plantes Cultivées, Montpellier, France; University of Manchester, United Kingdom

## Abstract

Approximate Bayesian Computation (ABC) has become a popular technique in evolutionary genetics for elucidating population structure and history due to its flexibility. The statistical inference framework has benefited from significant progress in recent years. In population genetics, however, its outcome depends heavily on the amount of information in the dataset, whether that be the level of genetic variation or the number of samples and loci. Here we look at the power to reject a simple constant population size coalescent model in favor of a bottleneck model in datasets of varying quality. Not only is this power dependent on the number of samples and loci, but it also depends strongly on the level of nucleotide diversity in the observed dataset. Whilst overall model choice in an ABC setting is fairly powerful and quite conservative with regard to false positives, detecting weaker bottlenecks is problematic in smaller or less genetically diverse datasets and limits the inferences possible in non-model organism where the amount of information regarding the two models is often limited. Our results show it is important to consider these limitations when performing an ABC analysis and that studies should perform simulations based on the size and nature of the dataset in order to fully assess the power of the study.

## Introduction

Central to evolutionary biology and science in general is the need to quantitatively compare models and hypotheses. In population genetics estimating parameters from more complex, biologically realistic models often involves a likelihood function that is difficult to compute. This has led to the development of methods, such as Approximate Bayesian Computation (ABC; [1]), that aim to approximate the likelihood function by simulating under a given model and using summary statistics to capture key aspects of the data in the most informative way (see [2] for an historical overview). Due to the flexibility and efficiency of ABC it is now possible to compare and estimate parameters from a number of complex models, and this has led to the widespread adoption of the method within the population genetics community for assessing and fitting demographic models to molecular data.

Understanding the evolutionary history of a population is an important aspect of studies on natural populations. Aside from giving information about the evolutionary past of organisms, inferring the demographic history and structure of a population is also necessary to understanding the effect of other population genetic processes. For instance, studies aiming to infer signatures of selection at candidate loci or across the genome depend on first knowing the background patterns of genetic variation produced by historical demographic events [3], [4]. Methods for estimating demographic histories have therefore become increasingly important, and have fuelled the proliferation of studies using ABC to infer a suitable demographic model.

A typical ABC workflow would consist of a number of steps: i) choose a set of summary statistics describing a given dataset; ii) perform a large number of simulations sampling a pre-supposed distribution of models and model parameters; iii) compute the summary statistics for the simulations; iv) apply a rejection threshold to focus on a region of the parameter space where the relationship between the summary statistics and parameters is assumed to be linear; v) perform either a regression to evaluate model parameters or perform a logistic regression to compare models. There are alternatives to this workflow, but this is the approach most commonly implemented in ABC analyses. The great strength of ABC lies in its flexibility, allowing the user to address a very large set of demographic models.

There are, however, a number of caveats associated with the approximation quality of ABC. These have been well-documented in the literature, but perhaps the most important consideration, and which is inherent to the ABC procedure, is in choosing informative summary statistics [5]–[7]. The field of population genetics has a long history of summarizing patterns of genetic variation in a way that is sensitive to departures from the standard neutral model. However, the extent to which summary statistics accurately represent the data is hard to evaluate and might be a major limitation to model inference, and particularly model choice [8]. This process has been relatively overlooked in the literature compared with advances in statistical methods that all somehow assume that data are properly summarized. Furthermore, even if a certain set of summary statistics is informative in accurately estimating parameters from two different demographic models separately, the same set of summary statistics may be uninformative when it comes to comparing these two models with each other [8].

Besides estimating demographic parameters such as population divergence times or migration parameters, model choice is central to many questions in population genetics. The problem of appropriately summarizing the data could be more important for datasets containing low levels of information, either because of an insufficient sampling effort or low levels of variation. Using population genetic simulations, we try to identify what happens when limitations are placed on the amount of information in the data, such as sample size, number of loci and level of genetic diversity. Firstly, this mimics many studies of natural populations where constraints are placed on the amount of data that can be collected. As the use of ABC has increased, so too has it been embraced in non-model organisms where the number of loci and samples are often limited. It is therefore of great interest to understand how a limited dataset impacts the use of ABC model choice. Secondly, it highlights which aspects of an ABC analysis, including the choice of summary statistics, are important in determining the power to reject a null demographic model in favor of a more complex alternative. Placing constraints on the data limits the amount of information available for comparing models, and by doing this we look to tease apart the factors contributing to the power of model choice in ABC.

Here, we use simulations to explore the power of model choice in ABC. In particular, we concentrate on two simple coalescent models commonly used in population genetic studies. The first is a null model of constant effective population size (Standard Neutral Model - SNM), and the second is a simple bottleneck model (BNM) that acts as our alternative model. Bottlenecks are known to occur frequently in natural populations (e.g. [9]–[12]) and are one of the most commonly investigated demographic models. There is considerable interest in understanding the patterns that bottlenecks leave in genetic data and a lot of work has gone into correctly inferring the parameters of bottleneck models in model species such as *Homo sapiens* and *Drosophila melanogaster* (reviewed in [13]). The model also contains a parameter controlling the severity of the bottleneck, and varying this parameter allows us to investigate the performance of model choice in ABC in more detail. We begin by exploring the relationship between the parameters of the models and a number of summary statistics commonly used in population genetics. Using a subset of these summary statistics, we assess the power to reject the SNM in favor of the BNM whilst varying: 1) the quality of the dataset; 2) the severity of the bottleneck; and 3) the tolerance of the rejection step.

## Results

### Choice of summary statistics

**Figure 1:**
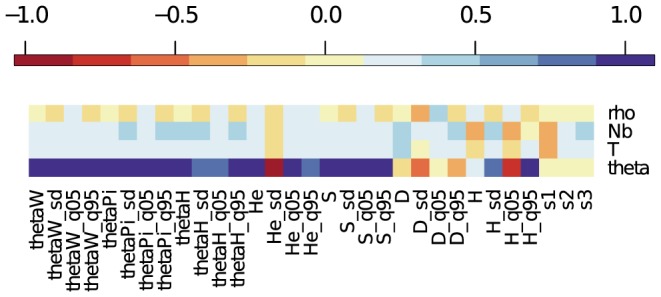
Correlation between parameters and summary statistics. Correlation coefficients between parameters and summary statistics for a SNM (top) and a BNM (bottom) for the larger dataset (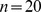, 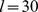). The parameters are given by theta and rho in the SNM and by theta, botEnd, botNe and rho in the BNM. The summary statistics give the average, standard deviation (_sd) and the 5 and 95% quantiles (_q5 and _q95) for three common estimates of 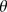 (thetaW, thetaPi, thetaH), haplotypic diversity (He), the number of segregating sites (S) and two tests of neutrality (Tajima's D and Fay & Wu's H). The site frequency spectrum is binned into 3 frequency classes (s1, s2, s3), ranging from low frequency (s1) to high frequency (s3) variants, that represent the proportion of segregating sites that occur in each class.

Figure 1 shows correlation coefficients between different summary statistics and the parameters of the SNM and BNM for the largest dataset with high genetic variance (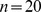, 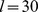, 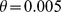). The parameter 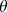 is strongly positively correlated with the means of many statistics, such as 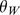, 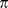, 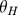, 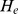 and 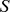, as well as their quantiles and standard deviations. One exception is the standard deviation of 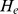 which is strongly negatively correlated with 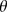. This is in sharp contrast with 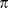 which responds positively to an increase in segregating sites. The standard deviation of Tajima's 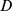 is negatively correlated with 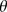, showing that its precision increases with increasing variation. The average value of Fay and Wu's 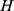 is independent of 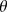 in the SNM and is slightly positively correlated with 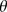 in the BNM. The variance of 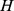 is strongly positively correlated with 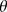, but the width of the interval between the 5% and 95% quantiles of 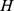 increases markedly with increasing 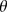, although this is due to the use of the non-standardized version of 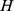. Finally, the site frequency spectrum is little affected by the mutation rate, save for a small positive correlation of s1 with the SNM, possibly due to an increased power of detection of rare variants with large values of 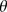. In contrast, 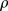 does not have any strong correlation with the means of the statistics. Its effect, however, is visible on the standard deviation, as recombination reduces the variance of the coalescent process. The mean of some statistics, such as Tajima's D, Fay & Wu's H and the site frequency spectrum appear independent of 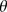, whereas the mean of 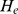 shows a strong correlation, reflecting an increase in the number of haplotypes with an increase in recombination. Parameters specific to the BNM (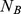 and 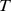) are both weakly correlated with most statistics, with the direction of the correlation consistent for both parameters. Both 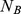 and 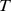 are most strongly correlated with Tajima's D (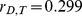, 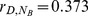), Fay & Wu's H (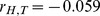, 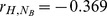) and the low frequency class of the site frequency spectrum (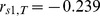, 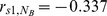). Among the three classes of the SFS (s1, s2 and s3) the proportion of low frequency variants (s1) is negatively correlated with both 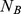 and 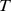. Interestingly, more recent and stronger bottlenecks (that is, low values for 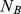 and 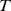) both result in negative 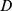 and an excess of rare variants with a corresponding depletion of high frequency classes. 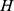 and its variance also respond to the bottleneck parameters, particularly its severity.

**Figure 2:**
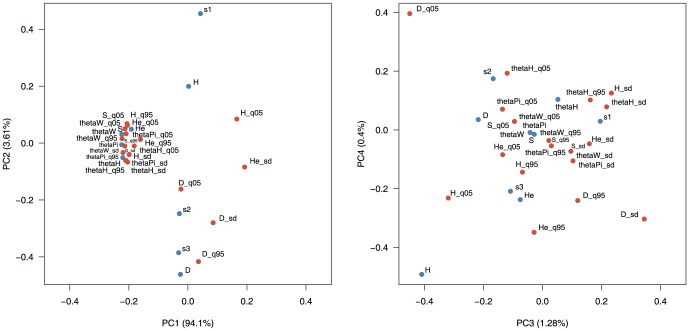
Principal Component Analysis of summary statistics under a bottleneck model. The first four principal components (PCs) of a PCA for summary statistics calculated for 10,000 simulations of a BNM in a large, genetically diverse sample (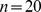, 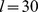, 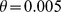).

We performed a Principal Component Analysis (PCA) in order to quantify the main features that can be extracted from summary statistics. PCA reduces the dimensions of a set of potentially correlated variables into a smaller set of uncorrelated variables that best explain the variance in the data. Figure S1 shows a PCA of the SNM (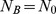, 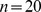, 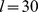, 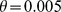) and the BNM (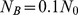, 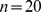, 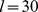, 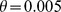) contrasted against the complete parameter space of the BNM (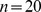, 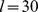). The two models are clearly separated on the first principal component (PC) indicating that there is enough information in the summary statistics to distinguish between the models. There is still a separation between the models on the second and third PCs, but they cannot be distinguished on the fourth PC. Figure 2 shows the first four PCs (PC1, PC2, PC3 and PC4) for the summary statistics calculated under a BNM. Each of the (independent) principal components can be linked to a feature of the genetic polymorphism patterns, and these features can be measured by one or more statistics. For example, PC1 represents the parameter 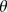 as many of the 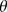 estimators cluster together and there is the same pattern of correlation with statistics as observed in Figure 1. The first PC captures most of the signal (94.1%), showing that 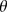, which controls the amount of genetic variation, is the major parameter shaping the patterns of polymorphism. The second PC captures far less of the signal (3.6%) and likely represents the shape of the site frequency spectrum, as there is differentiation among statistics known to be influenced by the shape of the site frequency spectrum. It is essentially independent from the first PC, and correlated with the three site frequency spectrum categories, the neutrality tests 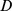 and 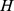 and the quantiles and standard deviation of 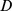 (those of 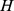 are strongly correlated with 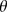). The low-frequency variants and Tajima's 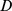 contribute most to this PC, although in opposite directions. The third and fourth PCs capture less of the variation (1.3% and 0.4% respectively) but there are still interpretable patterns in the data. In particular, 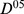, 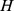 and 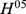 are clustered separately from the rest of the summary statistics on the third PC and are then separated on the fourth PC. It is possible that these represent derived alleles and that this could be informative for model choice.

### Comparison amongst sets of summary statistics

We looked at the effect that a 90% reduction in the effective population size (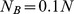) has on the power to reject the SNM for different sets of summary statistics (Table 1). All sets of summary statistics give good power for large datasets with high nucleotide diversity (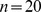, 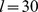, 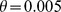), with the proportion of Bayes factors exceeding 3 all being greater than 0.99 (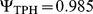; 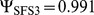; 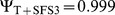; 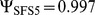; 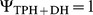). For a large dataset with low genetic diversity (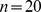, 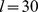, 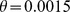), three sets of summary statistics are able to reject the SNM with a power greater than 0.9 (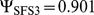; 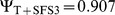; 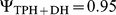), with TPH, in contrast, having very low power (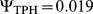). Smaller datasets afford less power to reject the SNM, except when using the TPH+DH set of summary statistics with high nucleotide diversity (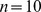, 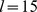, 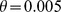), which still allows for a high degree of power in rejecting the SNM (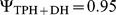).

**Table 1:** Power and false positive rate for different sets of summary statistics.

		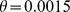					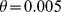				
		TPH	SFS3	T+SFS3	SFS5	TPH+DH	TPH	SFS3	T+SFS3	SFS5	TPH+DH
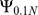	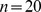 , 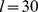	0.019	0.901	0.738	0.907	0.95	0.985	0.991	0.999	0.997	1
	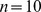 , 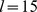	0	0.306	0.214	0.339	0.373	0.287	0.278	0.482	0.447	0.95
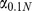	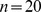 , 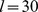	0	0.041	0.029	0.028	0.029	0.01	0.01	0.019	0.004	0.008
	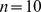 , 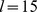	0	0.002	0.003	0.009	0.04	0.003	0.001	0.011	0	0.01

The power (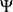) and false positive rate (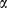) associated with rejecting a SNM in favor of a BNM for the TPH (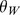, 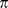, 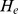), SFS3 (3 bin relative site frequency spectrum), T+SFS3 (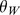, 3 bin relative site frequency spectrum), SFS5 (5 bin relative site frequency spectrum) and TPH+DH (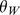, 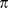, 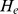, 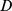, 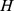) sets of summary statistics. 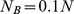.

Table 1 also shows the proportion of times that the SNM is falsely rejected (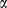). For three of the statistics (TPH, SFS3 and SFS5), the false positive rate is marginally higher in larger datasets, whereas for the TPH+DH set of statistics the pattern is the opposite. In small datasets, false positives are very rare (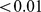) with a larger 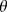 tending to decrease the rate of false positives. Different sets of summary statistics also result in different 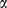: in particular, SFS3 tends to cause slightly more false positives than other sets of summary statistics. The number of false positives are small so any patterns may be influenced by the underlying variance. However, in none of the categories does the false positive rate reach 5%. While this is encouraging, it is important to note that the false positive rate increases as the Bayes factor used to determine significance is reduced. For large datasets with low genetic variation and the TPH+DH set of summary statistics the false positive rate is 0.166 and 0.397 for Bayes factor cutoffs of 1.5 and 1 respectively. Similarily, for small datasets with high genetic variation the rate is 0.110 and 0.411 for cutoffs of 1.5 and 1 respectively.

In general, the inclusion of statistics that summarize elements of the site frequency spectrum give greater power, which is most clearly reflected in smaller, low diversity datasets (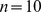, 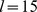, 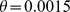) by the power difference between TPH and the other three sets of summary statistics (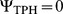; 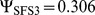; 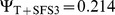; 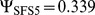; 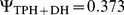). The distributions of model probabilities (Figure S2) under the SNM and BNM overlap for TPH, even for larger datasets where an increase in samples and loci leads only to a shift in the mean of the distribution so that the models are difficult to separate. However, for sets of summary statistics that incorporate the site frequency spectrum (TPH, SFS5 and TPH+DH) a larger dataset increases the power to distinguish between the two models, and the distribution of model probabilities is skewed.

**Figure 3:**
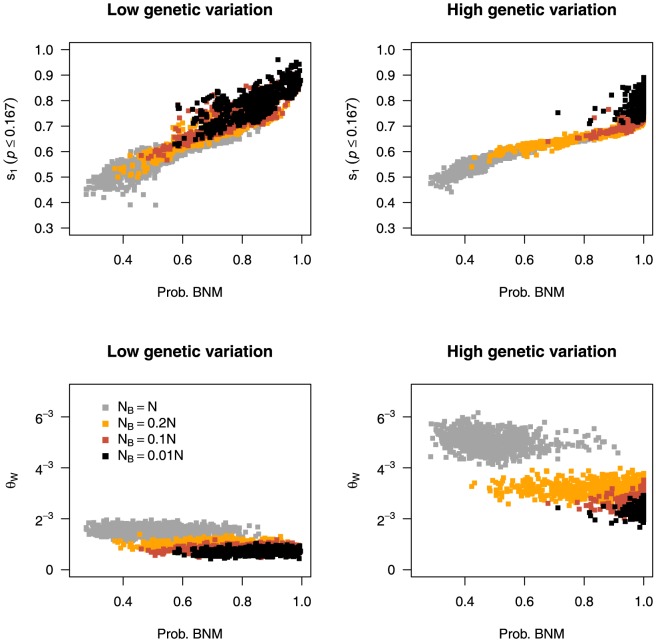
The impact of bottleneck severity and dataset quality on model probabilities and summary statistics. The effect of bottleneck strength on the value of summary statistics and model probabilities for larger datasets (n = 20, l = 30) with low (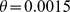) or high (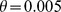) levels of genetic variation. Each point represents the rejection step of an ABC analysis when the T+SFS3 set of statistics is used with a tolerance of 0.001. The y-axis of the top two panels show the values of the first bin of the relative site frequency spectrum s1 (representing rare alleles) and the bottom panels display the value of Watterson's Theta (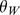). The effective population size during the bottleneck (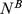) is defined relative to the recovered effective population size (N).

Somewhat surprisingly, including 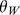 with SFS3 decreases the power for datasets with low genetic diversity (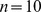, 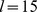: 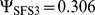, 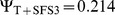; 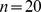, 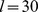: 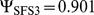, 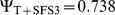). To investigate the performance of T+SFS3 in datasets with low and high levels of nucleotide diversity, we extended our analysis to include scenarios with more severe (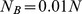) and less severe (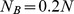) bottlenecks. In genetically diverse datasets there is a clear difference between the value of 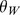 in BNMs and SNMs (Figure 3), suggesting that the statistic is informative in choosing between the models. In contrast, the distributions of 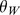 for datasets of low nucleotide diversity overlap to some extent, implying that in this case the statistic is not as informative for distinguishing between the two models.

In all scenarios considered, an increase in the severity of the bottleneck increases the power to correctly reject the SNM (Table 2). For all sets of summary statistics, there is excellent power to detect a 90% (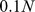) or 99% (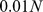) reduction in the effective population size with a large, genetically diverse sample (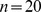, 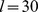, 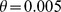). The choice of summary statistic becomes increasingly important for a smaller reduction in the population size of 80% as, even for large, genetically diverse samples (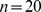, 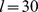, 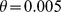), only the TPH+DH set of statistics performs well (0.968). The TPH set of statistics performs poorly with smaller or genetically less diverse datasets, even for strong bottlenecks (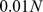), and seems to perform particularly badly in samples with low diversity.

**Table 2:** The effect of bottleneck severity on power.

		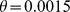			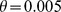		
		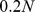	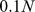	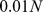	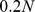	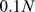	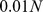
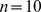 , 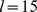	TPH	0	0	0	0.112	0.287	0.395
	SFS5	0.154	0.339	0.497	0.132	0.447	0.745
	T+SFS3	0.097	0.214	0.33	0.227	0.482	0.732
	TPH+DH	0.205	0.373	0.546	0.614	0.95	0.999
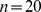 , 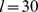	TPH	0.002	0.019	0.031	0.707	0.985	0.999
	SFS5	0.602	0.907	0.973	0.789	0.997	1
	T+SFS3	0.434	0.738	0.881	0.835	0.999	0.999
	TPH+DH	0.71	0.95	0.997	0.968	1	1

The power to correctly reject the SNM in favor of BNMs of different strengths, expressed as the relative effective population size during the bottleneck.

**Figure 4:**
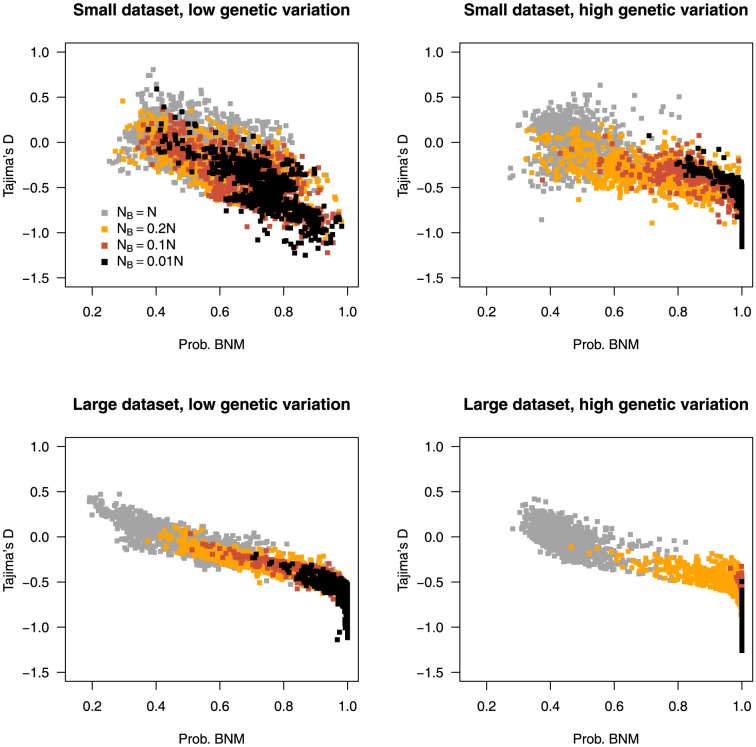
The impact of bottleneck severity and dataset quality on Tajima's D. The effect of bottleneck strength on the value of Tajima's D and model probabilities for both small (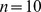, 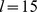) and large (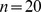, 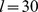) datasets with low (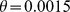) and high (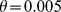) genetic variation. Each point represents the rejection step of an ABC analysis when the TPH+DH set of statistics is used with a tolerance of 0.001. The effective population size during the bottleneck (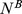) is defined relative to the recovered effective population size (N).

The TPH+DH set of statistics performs better than all other sets of statistics for all dataset types and bottleneck strengths tested. The power to detect population size reductions of 90% or more is greater than or equal to 0.95 for all but the worst datasets, whether that be a smaller dataset with higher genetic diversity (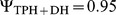; 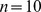, 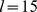, 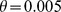), or a larger dataset with lower genetic diversity (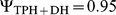; 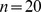, 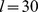, 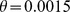). For smaller datasets with low genetic diversity, however, the power is still low to reject the SNM (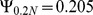; 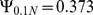; 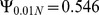). To dissect the performance of TPH+DH, we looked at the value of Tajima's D and Fay & Wu's H as a function of the model probabilities for bottlenecks of varying severity. The value of Tajima's D (Figure 4 and Figure S3) decreases with an increase in the severity of the bottleneck. For small datasets with low genetic variation (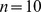, 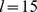, 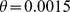), the overlap in values of Tajima's D is considerable, even between the SNM (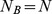) and the most severe bottleneck model (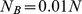), and this is in line with the low levels of power for this type of dataset (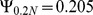; 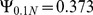; 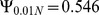). Larger datasets (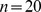, 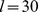) reduce the variation, whilst higher levels of genetic variation produce more negative mean values of Tajima's D (large dataset/low genetic variation: D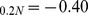, D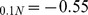, D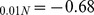; large dataset/high genetic variation: D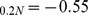, D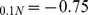, D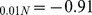). An increase in the severity of the bottleneck causes an overall increase in Fay & Wu's H (Figure S4). There is considerable difference between datasets of low and high nucleotide diversity in the variance of 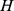. There is just a small shift in the mean of 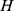 in datasets with low nucleotide diversity (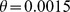) as the severity increases, suggesting that 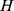 is uninformative. However, in datasets with high nucleotide diversity (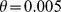) there is a larger increase in 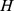 with increasing bottleneck severity.

**Figure 5:**
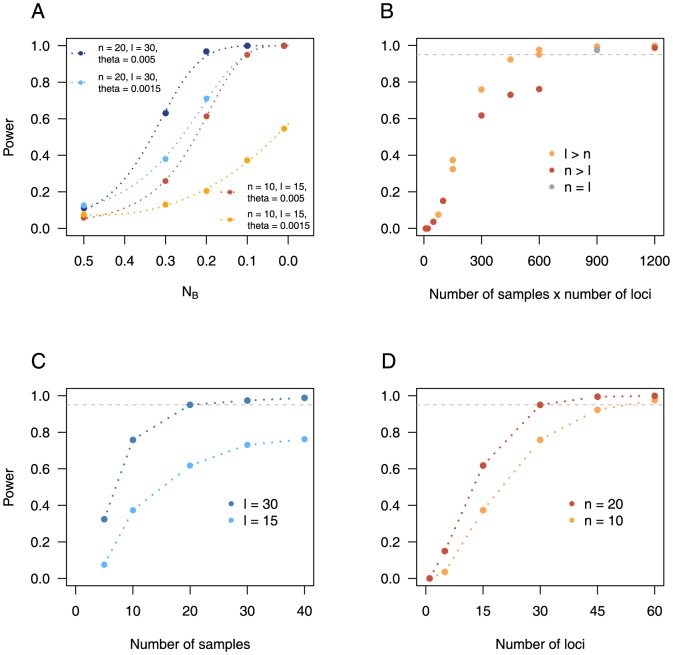
The effect of sample size and the number of loci on power. The power to reject the SNM, for the TPH+DH set of summary statistics, as a function of (A) the bottleneck severity (expressed as the relative effective population size 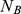), (B) the product of the number of loci and the number of samples (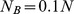, 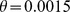), (C) the number of samples (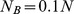, 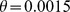), and (D) the number of loci (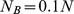, 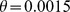). In B, C and D the dotted line corresponds to a power of 0.95.

For the most informative set of statistics (TPH+DH) we performed additional analyses for bottlenecks of different levels of severity (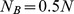 and 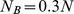) to better understand the relationship between bottleneck severity and the power to reject the SNM (Figure 5A). The power to detect weak bottlenecks (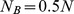) is low for each of the datasets tested. Contrastingly, for severe bottlenecks with a 99% reduction in the effective population size, all but the most limited datasets have very high power (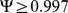). For a small dataset with low genetic diversity (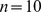, 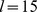, 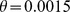), there is low power to reject the SNM for even the most severe bottlenecks (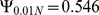).

### Varying the tolerance

We investigated the power of model choice in ABC when the tolerance was varied (Table S1 and Figure S5). Generally, a stricter tolerance leads to a higher level of power, especially for the TPH set of statistics, where the choice of tolerance is very important. For larger datasets with high nucleotide diversity (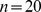, 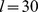, 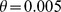), choosing a tolerance of 0.001 gives a power of 0.985 to reject the SNM, whereas using a tolerance level of 0.005 would result in a much lower power value of 0.525. Similarly, for large datasets with low nucleotide diversity (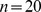, 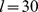, 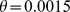) there is virtually no power to correctly reject the SNM, except when a tolerance of 0.0001 is used, which gives a power value of 0.688. However, a stricter tolerance leads to a higher rate of false positives, with rates reaching a maximum of 1.7% (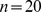, 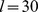, 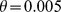, tol = 0.0001) and 3.2% (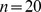, 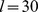, 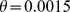, tol = 0.0001) for the TPH and SFS5 set of statistics respectively. The choice of tolerance appears to decrease in importance as the summary statistics capture more features of the site frequency spectrum. Tolerance levels of 0.01, 0.005 or 0.001 all give similar levels of power for the SFS5 set of summary statistics, with notable decreases being observed only with the most extreme tolerances (0.1 and 0.0001). It is worth noting however that these patterns might just represent random deviations caused by the low number of false positives and higher variance.

### Finding the optimal dataset size

In population genetic studies of natural populations there may be limitations on the quality of the dataset available for sampling. It is therefore of interest to ask how many samples or loci need to be obtained in order to have a 95% power to detect a bottleneck. We therefore extended our analyses, using the most informative set of summary statistics (TPH+DH), to include datasets with samples of between 5 and 40 individuals, where the number of loci varied between 1 and 60 and the level of genetic variation was low (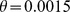). Figure 5B shows the power as a function of the product of the number of samples (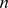) and loci (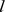) as this can be seen as being proportional to the sequencing cost of a study. The relationship between 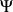 and 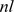 is sigmoidal, with the addition of loci and samples increasing the power above 95% when 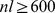. However, the contributions of the number of samples and loci to the power is not equal, with datasets having more power when 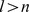.

Figure 5 also shows the power to reject the SNM in favor of a BNM for a different number of samples when the number of loci are limited (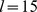 or 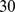; Figure 5C) and for a different number of loci when the number of samples is limited (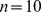 or 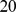; Figure 5D). When the number of loci are limited to 15 the power to reject the SNM remains well below 95%, even for large sample sizes (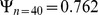), and the relationship appears to be asymptotic. Increasing the number of loci to 30 greatly increases the power such that sampling more than 20 individuals means that the power is greater than 95%. When the number of samples are limited 95% power is reached when 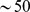 and 30 loci are used for sample sizes of 10 and 20 individuals respectively.

### Parameter estimation

**Figure 6:**
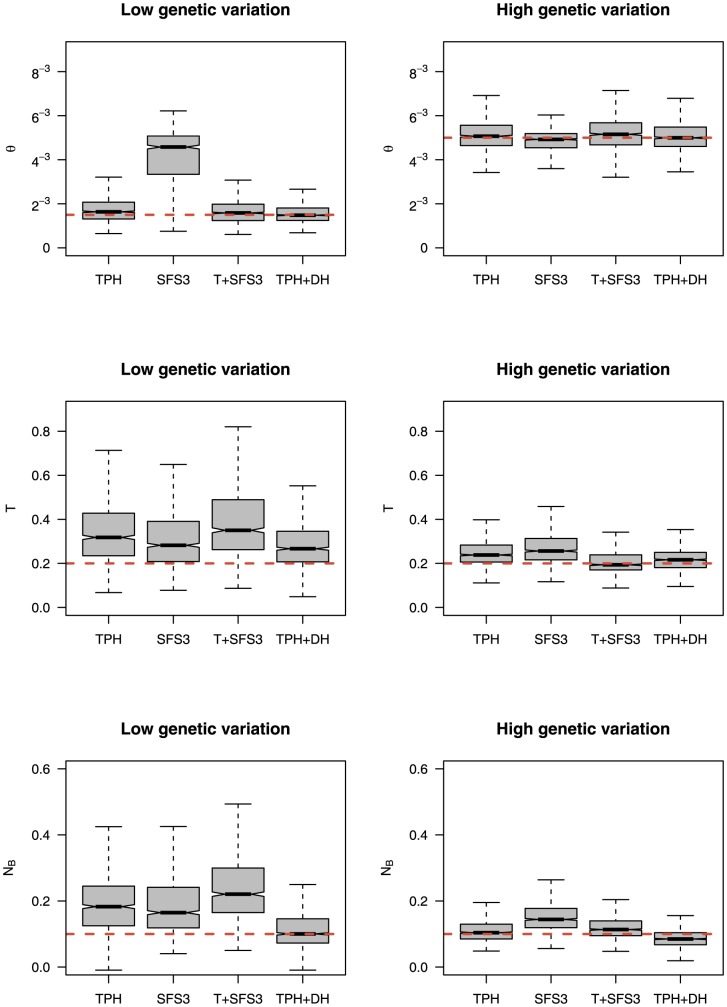
Parameter estimation for different sets of summary statistics. Boxplots showing the distribution of mean values of the 1000 posterior distributions for the replicates for the population scaled mutation rate (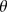), the time of the bottleneck (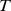) and the strength of the bottleneck (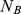) for datasets with low (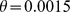) and high (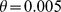) genetic variation. Thick lines denote the median, the boxes extend to the first (25%) and third quartiles (75%) and the whiskers give the minimum and maximum values. The dotted red lines show the true values for the BNM (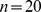, 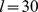).

To assess the ability of ABC to estimate parameters of a BNM in limited datasets, we performed parameter estimation using the local linear regression method described in [1]. Figure 6 and Table 3 summarize the distribution of the means of the posterior distributions for each replicate under a BNM for large datasets (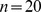, 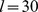). The parameter 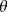 is estimated well by three of the four sets of summary statistics. SFS3, however, gives a very poor estimate of 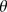 (0.00413) in low diversity samples, whilst the estimate is far better in high diversity samples (0.00475). The time of the bottleneck (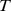) was estimated better in the high diversity samples than in the low diversity samples, with an increase in nucleotide diversity also decreasing the variance of the posterior means. The effective population size during the bottleneck (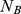) is estimated well for TPH+DH in both the low and high diversity datasets. For the rest of the sets of summary statistics, samples with high nucleotide diversity give better results. Table S2 shows the proportion of replicates where the true value lies within the 90%, 50% and 10% credible intervals of the parameter posterior distributions. One notable observation is that, for SFS3, the proportion of replicates in which the true value lies within the credible intervals is surprisingly high. This is particularly striking for high levels of variation and 10% credible intervals where a proportion of 0.578 was found for SFS3, compared to much smaller values for the other summary statistics [7]. It is also interesting to note that, for each parameter for TPH+DH, the proportion of replicates where the true value lies within the credible intervals is better for low nucleotide diversity (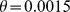) than for high nucleotide diversity (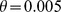).

**Table 3:** Parameter estimation under a bottleneck model.

	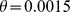			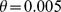		
	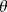 (0.0015)	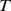 (0.2)	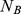 (0.1)	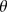 (0.005)	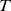 (0.2)	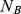 (0.1)
TPH	0.00173	0.34206	0.19495	0.00512	0.24841	0.11152
SFS3	0.00413	0.3143	0.18854	0.00475	0.27097	0.15404
T+SFS3	0.00166	0.38112	0.23845	0.00518	0.21601	0.12387
TPH+DH	0.00159	0.28889	0.11915	0.00507	0.21907	0.08665

Mean parameter estimates averaged over all replicates (true values in parentheses). 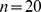, 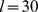.

## Discussion

In this study we chose to address the power of ABC model choice when the amount of data is the limiting factor. In ABC we are challenged with the task of summarizing the data such that these contrasting patterns are captured and there is enough information to distinguish between the two competing models. However, summary statistics that are capable of separating, for example, a population genetic model of constant effective population size from a population expansion model will not be the same as those capable of separating a structured population from an unstructured population [14]. Therefore, the way in which the data can be summarized most informatively will be highly context-dependent [15]. We began by first exploring the behaviour of summary statistics in a bottleneck model, and proceeded by investigating the power that different sets of summary statistics have in separating a bottleneck model from a simple model of constant effective population size.

### Choosing summary statistics

A number of studies (e.g. [16], [17]) have used correlation coefficients and PCA to guide their choice of summary statistics. We find that when using PCA it was possible to identify categories of summary statistics that are informative for separating the SNM and BNM. On the first PC, statistics strongly correlated with 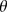 are separated from those based on the shape of the site frequency spectrum. This is in agreement with the finding that the inclusion of SFS-based statistics increases the power to reject the SNM in favor of a BNM. This is in line with population genetic expectations, a population bottleneck affects the average number of nucleotide differences more strongly than the number of segregating sites [18], and is the basis for Tajima's D. Tajima's D proved to be an effective and informative summary of the site frequency spectrum, becoming more negative with an increase in the severity. There may also be great benefit in using the unfolded site frequency spectrum. Only the folded site frequency was tested here, but any additional information given by knowing the derived allele could boost the power.

Additional signals in the third and fourth PCs indicate that derived alleles describe some of the variation, even if these PCs accounted for a small fraction of the overall variation (about 1.7% combined). Combining the signals from both low frequency and high frequency variants proved successful in increasing the power to reject the SNM. For example, of all the summary statistics tested, TPH+DH gave the highest power, and it may be that the combination of Tajima's D and Fay & Wu's H summarizes the site frequency spectrum in an informative way. Fay & Wu's H is often neglected as inferring the derived allele depends on there being a suitable outgroup available, which is not always the case in non-model organisms. In contrast, haplotypic information didn't appear to be overally informative, although this could simply be due to the size of the fragments simulated (750 bp). For the power analysis we assessed only the means of the statistics, but there may be information in the standard deviation and the quantiles of some of the summary statistics. In particular, the standard deviation of the haplotypic diversity showed a negative correlation with parameters of the BNM model.

One of the key considerations when choosing summary statistics has been in avoiding the curse of dimensionality [1]. As the number of summary statistics increases, so too does the variability in the parameter estimates in the regression step of ABC, leading to poorer parameter estimates. It has been suggested that model choice may be affected less by this problem [19]. While our results generally support this, we do find some limited evidence to the contrary in datasets with low genetic diversity where the power is lower for T+SFS3 compared to the SFS3 set of summary statistics. Accordingly, a number of methods have been established for identifying informative summary statistics in relation to estimating parameters. For example, [20] weight statistics according to the information they give for a parameter of interest, whilst [21] implement a partial least-squares transformation of the summary statistics and [22] apply a machine learning technique (boosting) to find the most informative summary statistics. More recently, research has moved towards identifying summary statistics for model choice. [19] use logistic discriminant analysis to process summary statistics before model choice, [23] weight the summary statistics for model choice after a preliminary regression step and [24] derive conditions under which summary statistics are sufficient for selecting the true model.

While we find PCA to be a highly informative way of summarizing the data, it may not be enough to simply perform PCA and look for patterns in the summary statistics. Some results of our analyses were counter-intuitive, such as the finding that the T+SFS3 set of summary statistics performed worse than SFS3 in datasets with low genetic diversity. This suggests that there can be a complex relationship between some summary statistics and the parameters of the model. This may be the case in the above example where Watterson's 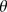 only adds noise rather than any additional information to the low diversity datasets and could, as discussed above, be due to the curse of dimensionality. However, the SFS3 set of statistics estimates the parameter 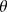 poorly. This may be due to the use of the relative instead of the absolute site frequency spectrum, as information in the absolute SFS regarding 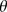 is lost when it is re-scaled. This draws to attention an interesting problem concerning the choice of summary statistics in ABC. A set of summary statistics that is informative in distinguishing between two competing models may not be the set of statistics most suitable for estimating parameters under the most probable model. In some cases it may be more suitable to perform model choice using a set of summary statistics known to be informative in separating a class of models, and then to use a second set of summary statistics for estimating parameters known to be informative for the most probable model. Foremost, this emphasizes the importance of performing preliminary analyses of the power afforded by a given set of summary statistics.

Whilst an important aspect to consider, our results suggest that the choice of tolerance is not of overriding importance, although this is dependent on how informative the summary statistics are. This has also been observed in other studies looking at parameter estimation in an ABC framework. [20], for example, found that the tolerance has a relatively minor effect on the estimation of migration rate when they weight summary statistics according to how informative they are. However, [25] noted that, in general a low tolerance was more beneficial for estimating parameters if the number of accepted replicates was sufficiently high. Of more importance is the choice of Bayes factor cutoff. In this study we used a Bayes factor of 3 as the threshold but note that the false positive rate increases with a decreasing Bayes factor cutoff. This suggests that drawing conclusions from analyses where Bayes factors are less than 3 may lead to the inference of an incorrect model.

### ABC in non-model organisms

The amount of data available for genetic studies of non-model organisms is often limited, and so it is important that sequencing and sampling efforts are directed towards maximizing the amount of information available for ABC. Specifically, we find that there are a number of factors that govern the power to distinguish between two competing models. In particular, the level of genetic variation is important and this inevitably has consequences on the number of loci and samples required. Here, we considered the power afforded to sequence data, but other types of markers, such as microsatellites, are more variable and would give more information. For our low variation datasets (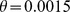), around 20 individuals and 30 loci would be required in order to have a 95% power to detect a strong bottleneck (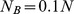). In the more variable dataset (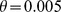), around 10 individuals and 15 loci would give the same power. In general, and in agreement with expectations of the coalescent [26], we find that sampling more loci rather than individuals is of greater benefit in increasing the power.

It is also important to acknowledge that there is a limit to what one can say with a limited dataset. Our analyses dealt with relatively strong bottlenecks, and these represent quite drastic demographic events. Weaker events will undoubtedly affect genetic data in a subtler way that is harder to detect and therefore requires more data, whether that be more loci, individuals or more variable markers. However, in general we find that the power to detect a bottleneck increases with the severity of the bottleneck. Large samples of loci and individuals are required to detect mild bottlenecks and this is likely to generalize to parameters in other models. Similarly, [27] found that there is lower power to detect a weaker migration rate in an isolation-with-migration model. However, even if bottlenecks with a reduction in the population size of 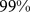 or more can de detected with the correct summary statistics, it is unclear how often bottlenecks of this magnitude occur in natural populations. There are examples (e.g. [9]: 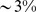 of current size; [28]: 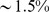 of current size), especially in domesticated species (e.g. [29]: 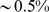 of current size), where drastic reductions in the effective population size have been inferred. However, it may be the case that weaker bottlenecks, or more subtle temporal variation in the effective population size, are more frequent but that we simply do not have enough power to detect them with the datasets at hand.

In general, the approach of ABC in summarizing the data into summary statistics is relatively reliable for estimating parameters [8]. Although this seems to be true in most cases, we find that this does not hold in cases where the summary statistics are less informative. This is exemplified by the poor estimate of 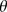 by the SFS3 set of summary statistics. This appears to be resulting from the priors, that are uniformly distributed between 0 and 0.01. When the summary statistics offer no information on the parameters of interest then the expected value of the parameter will be the mean of the prior distribution. In this case, the parameter estimate of 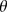 would approach 0.005 as the summary statistics become less informative, and would explain the poor estimate of 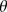 in low diversity samples (0.00413). A sensitivity analysis might therefore be an important step in determining the influence of the prior over the posterior distribution. This may also be influencing the other parameters. 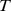 and 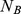 appear to be overestimated when the data or summary statistics are insufficient, and so these parameters could tend to the mean of the prior distribution if the dataset or summary statistics are insufficient. There are a number of ways that the estimation of parameters and model choice can be improved. The euclidean distance metric is most commonly used for assessment of the fit of the simulated data to the observed data, but other metrics may provide a better measure. Another common step is to even out the contribution of each of the summary statistics through a normalization step that conforms them to the same standard deviation, and this could lead to improvements in parameter estimation.

To assess the amount of information that the analysis brings, it is strongly advised that the prior and posterior distributions are compared, and this can identify situations where the prior has too much influence over the analysis. In the present study we have considered two simple models (SNM and BNM) and estimated our power to distinguish those. For both models the data were generated by the models under comparison. In real life, however, we may, for instance, sample individuals from different demes in a structured population. This can have a confounding effect and may lead to the false detection of bottlenecks or effective population size changes [30], [31].

## Conclusions

Our analysis of the power of ABC model choice in limited datasets suggests that careful consideration of the number of loci and samples is critical when designing a study. Even in scenarios as simplistic as the one examined here, under some conditions, there is simply not enough information contained in the data to confidently separate two distinct models. While ABC in principle allows testing for very complex demographic histories, the amount of information that can be extracted from a given dataset is likely to limit power to make more subtle inferences. However, certain parameters, such as the number of samples, the number of loci and the level of genetic variation, can be used fairly reliably to predict the power of a study to separate different models. What's more, if suitably informative summary statistics are used together with an appropriately large dataset then, in general, ABC model choice is relatively powerful and quite conservative with regard to the false positive rate. Fortunately, with the widespread availability of simulation tools, it is possible to test the probability of detecting a model with a dataset of any given size and level of diversity. Furthermore, the efficiency and flexibility of ABC means that assessing the power of any given dataset is realistic for most studies in non-model organsisms.

## Materials and Methods

### Demographic models and simulated datasets

We test the power of ABC by comparing two simple population genetic models, each simulated under a coalescent model. The coalescent is a backward in time simulator of a population of gametes that can be subjected to a number of evolutionary forces. The genetic variation in a population of gametes is determined by the mutation rate per generation (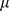), and the effective population size (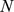). The level of genetic variation in a population is then defined as the product of these two parameters: 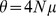. Two coalescent models were considered, the first of which consisted of a population of effective size 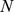 (haploid individuals) that remain constant through time (SNM). For the second scenario, a bottleneck model (BNM) was considered where an instantaneous reduction in the population size occurs at 0.2 coalescent time units (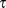) in the past (measured in 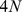 generations), and persists for a period of 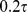 before returning to its original size. For each demographic model, 1000 datasets were simulated whereby the levels of nucleotide diversity (assuming an infinite sites model) and number of samples and loci were varied. For the majority of analyses, we considered a sample size (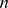) of 10 or 20 individuals (where a population consists of 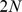 gametes), with 15 or 30 loci sequenced (750 bp each in length) and two levels of genetic variation with per base pair scaled mutation rates of 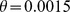 or 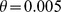 (where 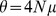). For the majority of simulations in the BNM, the relative population size during the bottleneck (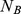) was 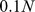, although we also varied this parameter (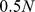, 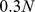, 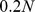, 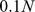 and 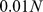) to assess the performance of ABC in detecting bottlenecks of varying severity. The population scaled recombination rate, 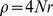, was set to 0.01/bp in each model.

For each of these simulated datasets the mean, standard deviation and 5% and 95% quantiles across loci were calculated for Watterson's 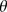 (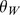, 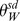, 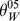, 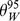; [32]), nucleotide diversity (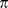, 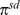, 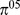, 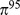; [33]), Fay & Wu's estimate of 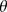 (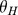, 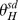, 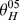, 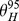; [34]), haplotype diversity (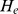, 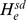, 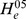, 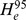; [35]), Tajima's D (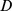, 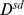, 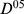, 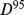; [36]), Fay & Wu's non-standardized H (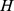, 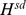, 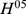, 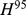; [34]) and the number of segregating sites (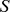, 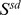, 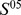, 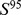). The relative site frequency spectrum (SFS) was also summarized by the average proportion of segregating sites that occur within each of three or five evenly sized frequency classes (s1, s2, s3, s4, s5). These represent population genetic statistics that are thought to summarize population sequence data in the most informative way (see for example, [17], [25], [26]). For the analysis of the power and false positive rate of ABC we combined a number of summary statistics: TPH (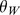, 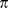, 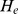), SFS3 (folded site frequency spectrum in 3 bins), T+SFS3 (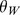, folded site frequency spectrum in 3 bins), SFS5 (folded site frequency spectrum in 5 bins) and TPH+DH (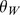, 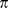, 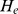, 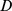, 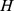). When analyzing the relationships among summary statistics, the calculation of correlation coefficients and the performance of Principal Component Analysis (PCA) was implemented using the python library NumPy.

### Model choice, power and parameter estimation

Joint posterior densities were simulated for each of the two ABC models using 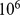 draws from uniformly distributed priors. The prior bounds for the SNM and BNM were (0, 0.01) and (0, 0.02) for 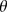 and 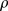, respectively. Time was measured on a scale of 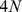 generation. For the BNM, the time (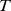) of the bottleneck (looking backwards in time) was sampled from a prior with bounds (0, 1.5), with the relative population size during the bottleneck (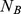) having bounds of (0, 1). The relative ancestral population size and the duration of the bottleneck were fixed as 1 and 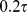 respectively. ABC was performed using the python library EggLib [37], which implements the Euclidean distance-based, local linear regression method described in [1]. Model choice was performed using the rejection-based method implemented in EggLib, with model probabilities being defined as the proportion of simulations belonging to each model after the ABC rejection step. Bayes factors were calculated as the ratio of the model probabilities, with a Bayes factor 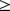 3 (unless stated otherwise) being considered an acceptable level of significance [38]. Power (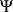) was defined as the probability of correctly rejecting the SNM and was assessed by calculating the proportion of replicates with a Bayes factor 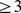 when the true model was the BNM. False positives were considered as instances where the SNM was falsely rejected and was given by the proportion of replicates with Bayes factors 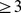 when the SNM was the correct model. Parameter estimation was carried out using the local linear regression method of [1]. The accuracy of parameter estimation was assessed by comparing the true parameter value with that estimated in ABC using the relative bias, 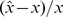, and the relative mean square error, 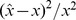. The tolerance level (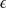) for both model choice and parameter estimation was fixed at 0.001 unless otherwise stated. Coalescent simulations, ABC analyses and calculation of summary statistics were performed using EggLib. Any additional custom code is provided in the Github repository: https://github.com/mspopgen/Stocks2014a.

## Supporting Information

Figure S1
**Principal Component Analysis under the SNM and BNM models.** The first four principal components (PCs) for summary statistics calculated under the SNM and a BNM (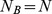, 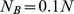) and the entire prior parameter space of the BNM. 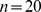, 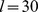.(PDF)

Figure S2
**Model probability distributions for different summary statistics.** Distribution of model probabilities for the TPH (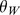, 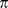, 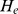), SFS5 (5 bin relative site frequency spectrum) and TPH+DH (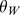, 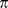, 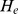, 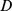, 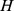) sets of summary statistics. 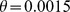, 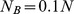.(PDF)

Figure S3
**Impact of bottleneck severity on Tajima's D.** The effect of bottleneck strength on the value of Tajima's D for both small (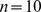, 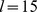) and large (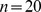, 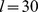) datasets with low (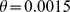) and high (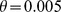) genetic variation. Each point represents the rejection step of an ABC analysis when the TPH+DH set of statistics is used with a tolerance of 0.001. The effective population size during the bottleneck (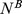) is defined relative to the recovered effective population size (N).(PDF)

Figure S4
**Impact of bottleneck severity on Fay and Wu's H.** The effect of bottleneck strength on the value of Fay & Wu's H and model probability for both small (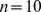, 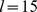) and large (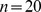, 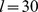) datasets with low (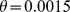) and high (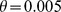) genetic variation. Each point represents the rejection step of an ABC analysis when the TPH+DH set of statistics is used with a tolerance of 0.001. The effective population size during the bottleneck (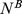) is defined relative to the recovered effective population size (N).(PDF)

Figure S5
**Impact of tolerance.** The effect of the tolerance level on model comparison in ABC for datasets with different numbers of samples (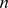), loci (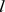) and levels of genetic variation. Different colored lines refer to different sets of summary statistics: TPH and SFS5. 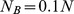.(PDF)

Table S1
**Impact of tolerance.** The power (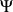) and false positive rate (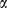) for different tolerances and sets of summary statistics. 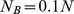.(PDF)

Table S2
**Parameter estimates.** Proportion of replicates where the true value lies within the 10%, 50% and 90% confidence intervals of the posterior distribution. 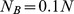, 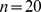, 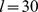.(PDF)
