# Supplementary figures and images for: Amount of Information Needed for Model Choice in Approximate Bayesian Computation
Source: PLoS One. 2014 Jun 24;9(6):e99581. doi: 10.1371/journal.pone.0099581 (PMC4069000; doi:10.1371/journal.pone.0099581)

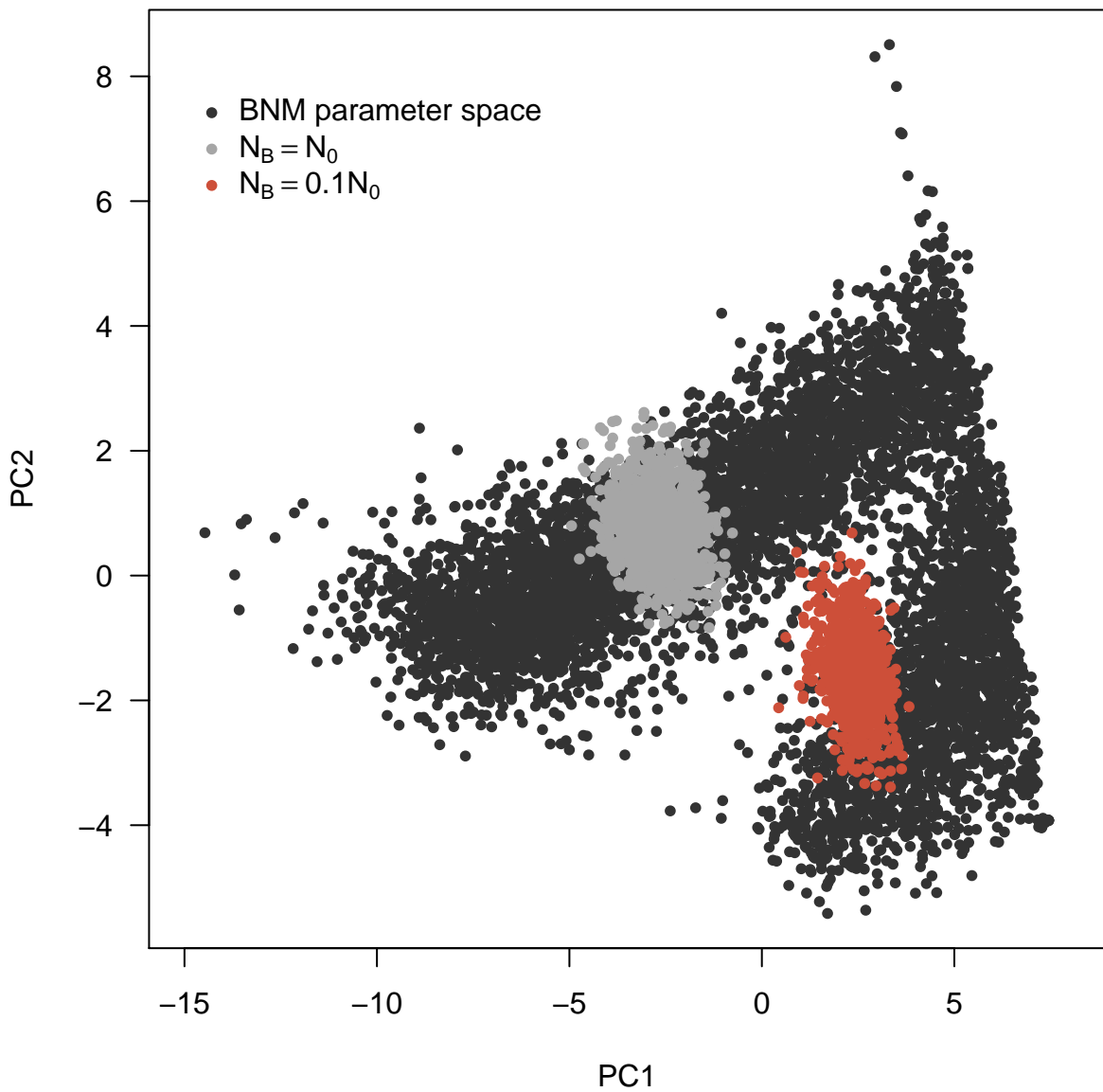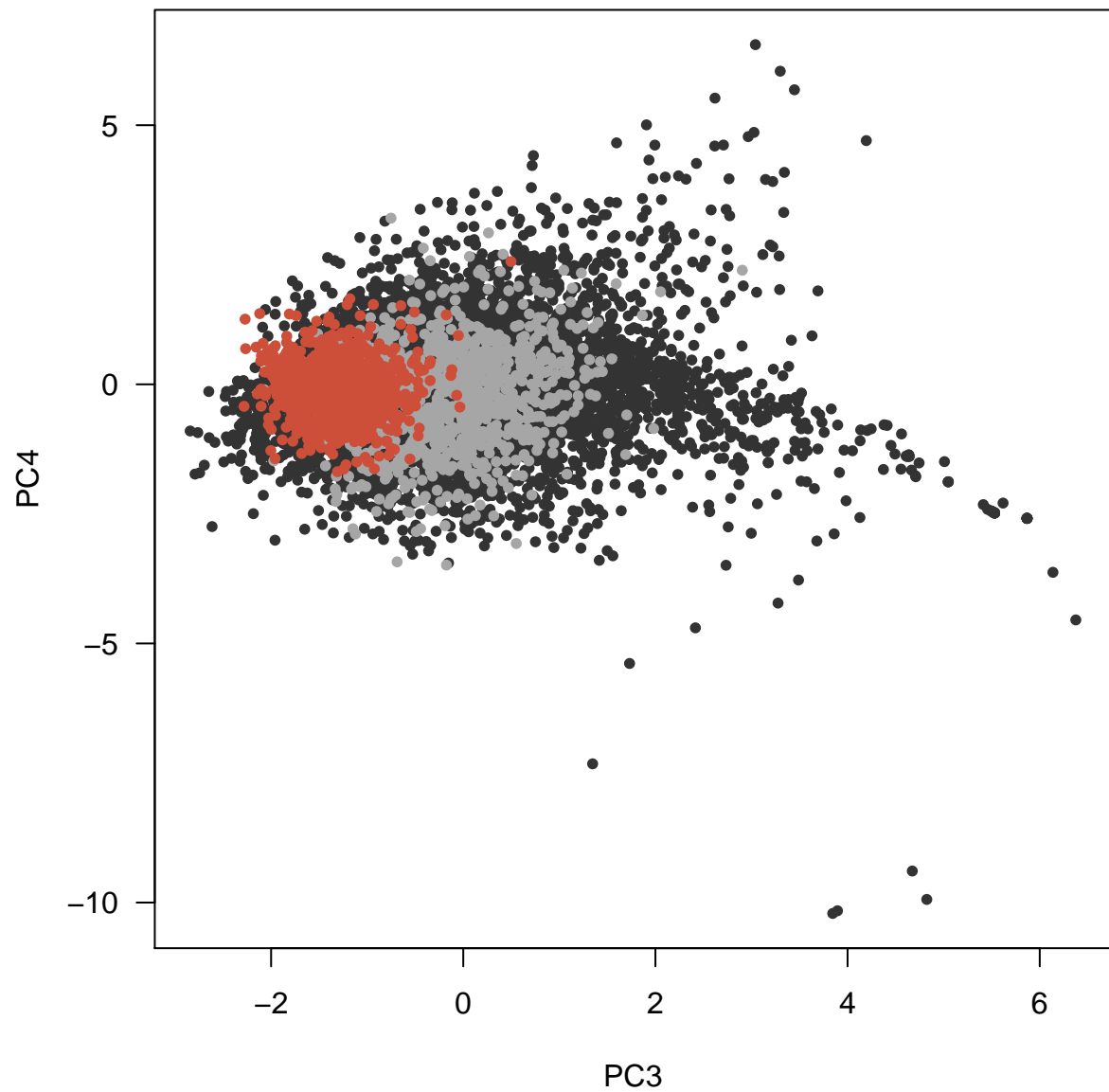

Supplement: Figure S1 — Principal Component Analysis under the SNM and BNM models. The first four principal components (PCs) for summary statistics calculated under the SNM and a BNM (, ) and the entire prior parameter space of the BNM. , . (PDF) [file pone.0099581.s001.pdf]

TPH,  $n = 10, l = 15$ 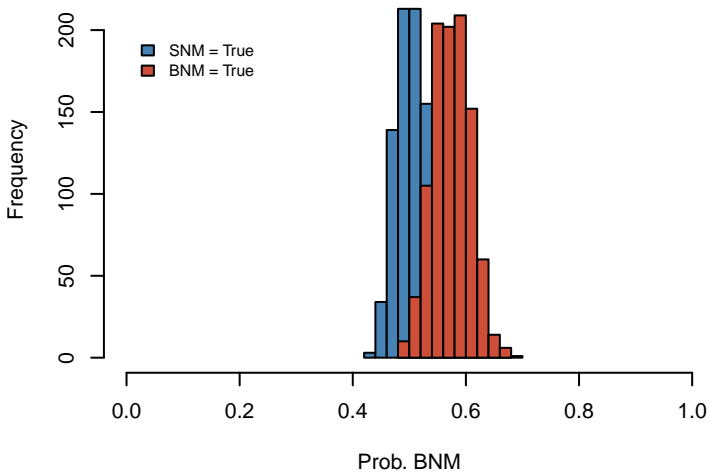TPH,  $n = 20, l = 30$ 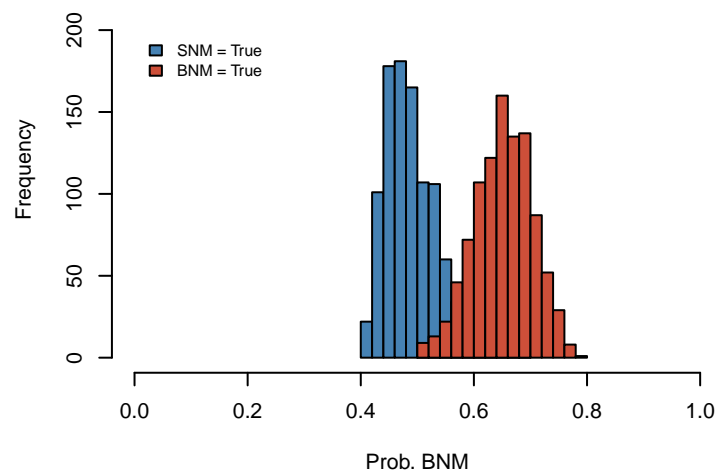SFS<sub>5</sub>,  $n = 10, l = 15$ 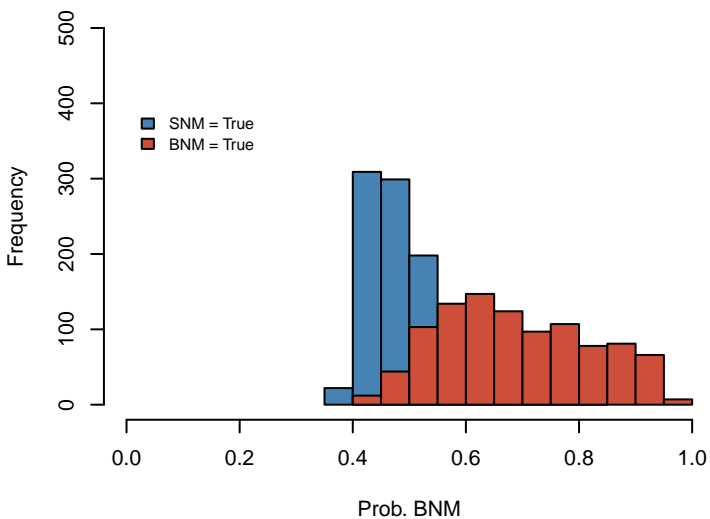SFS<sub>5</sub>,  $n = 20, l = 30$ 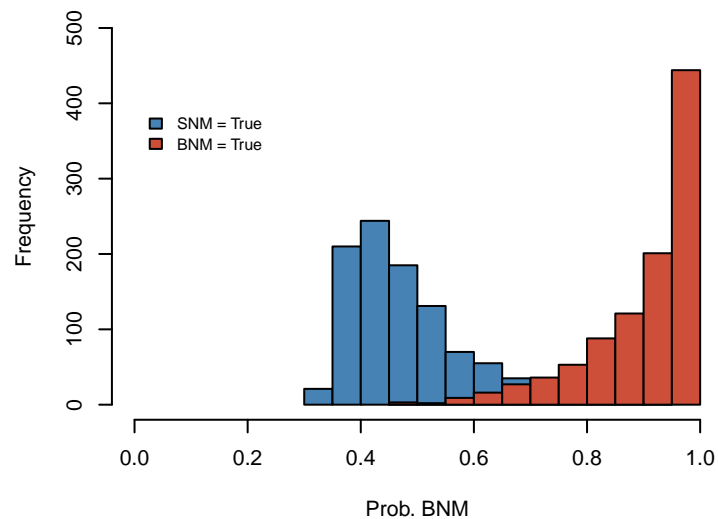TPH+DH,  $n = 10, l = 15$ 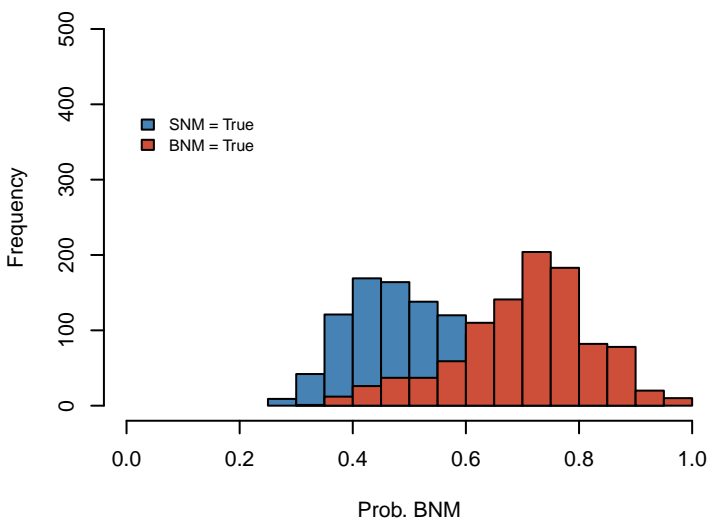TPH+DH,  $n = 20, l = 30$ 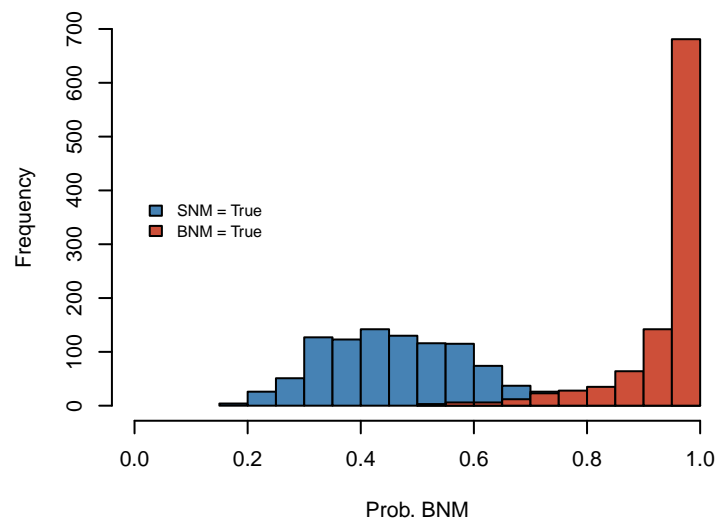

Supplement: Figure S2 — Model probability distributions for different summary statistics. Distribution of model probabilities for the TPH (, , ), SFS5 (5 bin relative site frequency spectrum) and TPH+DH (, , , , ) sets of summary statistics. , . (PDF) [file pone.0099581.s002.pdf]

**Power**  
**theta = 0.0015**

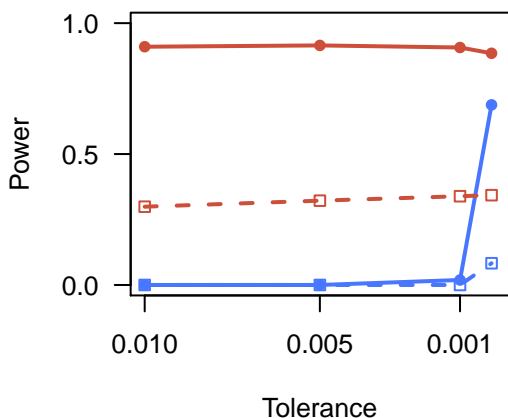

**False positives**  
**theta = 0.0015**

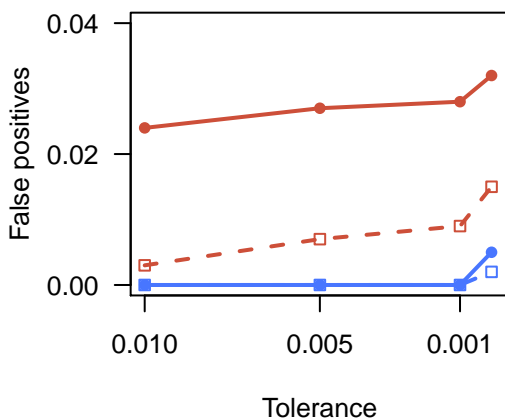

**Power**  
**theta = 0.005**

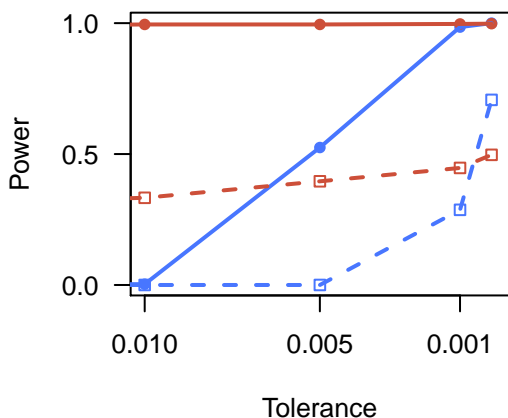

**False positives**  
**theta = 0.005**

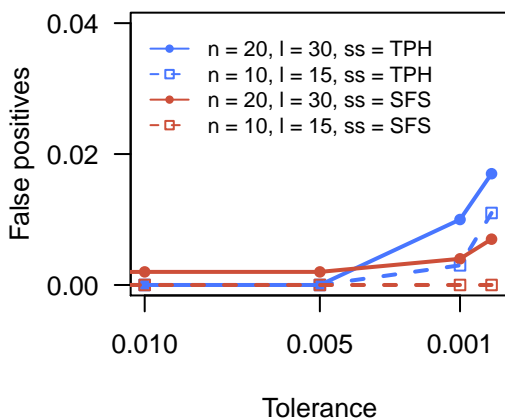

Supplement: Figure S5 — Impact of tolerance. The effect of the tolerance level on model comparison in ABC for datasets with different numbers of samples (), loci () and levels of genetic variation. Different colored lines refer to different sets of summary statistics: TPH and SFS5. . (PDF) [file pone.0099581.s005.pdf]
