# Supplementary material for: Amount of Information Needed for Model Choice in Approximate Bayesian Computation
Source: PLoS One. 2014 Jun 24;9(6):e99581. doi: 10.1371/journal.pone.0099581 (PMC4069000; doi:10.1371/journal.pone.0099581)
Supplement: Figure S3 — Impact of bottleneck severity on Tajima's D. The effect of bottleneck strength on the value of Tajima's D for both small (, ) and large (, ) datasets with low () and high () genetic variation. Each point represents the rejection step of an ABC analysis when the TPH+DH set of statistics is used with a tolerance of 0.001. The effective population size during the bottleneck () is defined relative to the recovered effective population size (N). (PDF) [file pone.0099581.s003.pdf]

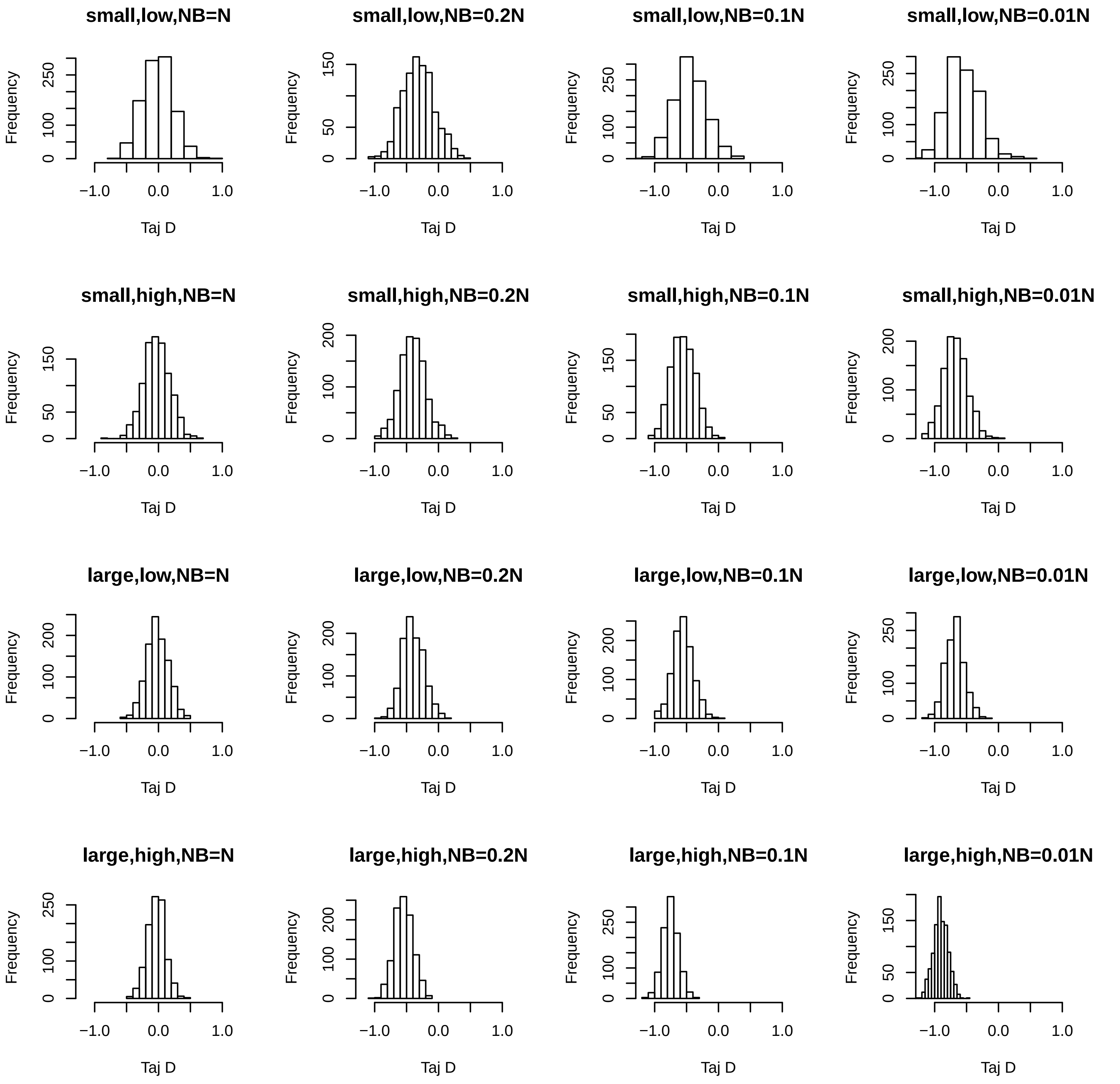
small,low,NB=N
small,low,NB=0.2N
small,low,NB=0.1N
small,low,NB=0.01N
small,high,NB=N
small,high,NB=0.2N
small,high,NB=0.1N
small,high,NB=0.01N
large,low,NB=N
large,low,NB=0.2N
large,low,NB=0.1N
large,low,NB=0.01N
large,high,NB=N
large,high,NB=0.2N
large,high,NB=0.1N
large,high,NB=0.01N
Frequency
Taj D
−1.0
0.0
1.0
