# Supplementary material for: Amount of Information Needed for Model Choice in Approximate Bayesian Computation
Source: PLoS One. 2014 Jun 24;9(6):e99581. doi: 10.1371/journal.pone.0099581 (PMC4069000; doi:10.1371/journal.pone.0099581)
Supplement: Figure S4 — Impact of bottleneck severity on Fay and Wu's H. The effect of bottleneck strength on the value of Fay & Wu's H and model probability for both small (, ) and large (, ) datasets with low () and high () genetic variation. Each point represents the rejection step of an ABC analysis when the TPH+DH set of statistics is used with a tolerance of 0.001. The effective population size during the bottleneck () is defined relative to the recovered effective population size (N). (PDF) [file pone.0099581.s004.pdf]

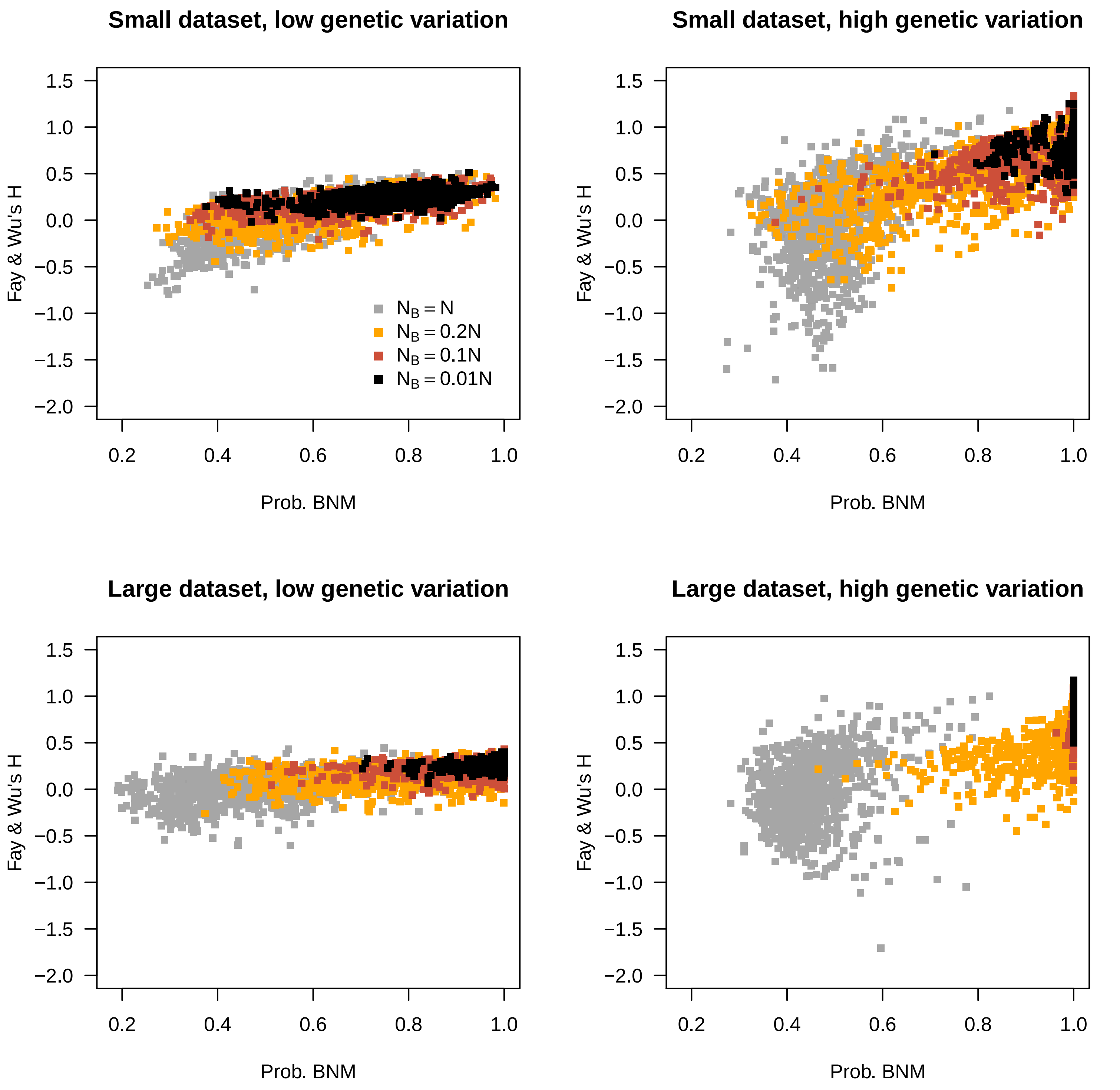

Small dataset, low genetic variation
Small dataset, high genetic variation
Large dataset, low genetic variation
Large dataset, high genetic variation
Fay & Wu's H
Prob. BNM
1.5
1.0
0.5
0.0
−0.5
−1.0
−1.5
−2.0
0.2
0.4
0.6
0.8
1.0
$N_B = N$
$N_B = 0.2N$
$N_B = 0.1N$
$N_B = 0.01N$
