# Supplementary material for: Amount of Information Needed for Model Choice in Approximate Bayesian Computation
Source: PLoS One. 2014 Jun 24;9(6):e99581. doi: 10.1371/journal.pone.0099581 (PMC4069000; doi:10.1371/journal.pone.0099581)
Supplement: Table S1 — Impact of tolerance. The power () and false positive rate () for different tolerances and sets of summary statistics. . (PDF) [file pone.0099581.s006.pdf]

Table S1. Impact of tolerance. The power (Ψ) and false positive rate (α) for different tolerances and sets of summary statistics. $N_B = 0.1N$.

			$\theta = 0.0015$					$\theta = 0.005$				
			0.1	0.01	0.005	0.001	0.0001	0.1	0.01	0.005	0.001	0.0001
$\Psi_{0.1N}$	TPH	$n = 20$, $l = 30$	0	0	0	0.019	0.688	0	0.003	0.525	0.985	1
		$n = 20$, $l = 15$	0	0	0	0	0.321	0	0.003	0.15	0.809	0.948
		$n = 10$, $l = 30$	0	0	0	0	0.234	0	0	0.003	0.7	0.956
		$n = 10$, $l = 15$	0	0	0	0	0.083	0	0	0	0.287	0.707
	$\mathtt{SFS}_5$	$n = 20$, $l = 30$	0.654	0.91	0.915	0.907	0.885	0.886	0.995	0.995	0.997	0.998
		$n = 10$, $l = 15$	0	0.299	0.322	0.339	0.343	0	0.333	0.396	0.447	0.497
$\alpha_{0.1N}$	TPH	$n = 20$, $l = 30$	0	0	0	0	0.005	0	0	0	0.01	0.017
		$n = 20$, $l = 15$	0	0	0	0	0.005	0	0	0	0.006	0.014
		$n = 10$, $l = 30$	0	0	0	0	0.006	0	0	0	0.002	0.008
		$n = 10$, $l = 15$	0	0	0	0	0.002	0	0	0	0.003	0.011
	$\mathtt{SFS}_5$	$n = 20$, $l = 30$	0.002	0.024	0.027	0.028	0.032	0	0.002	0.002	0.004	0.007
		$n = 10$, $l = 15$	0	0.003	0.007	0.009	0.015	0	0	0	0	0
