# Supplementary material for: Amount of Information Needed for Model Choice in Approximate Bayesian Computation
Source: PLoS One. 2014 Jun 24;9(6):e99581. doi: 10.1371/journal.pone.0099581 (PMC4069000; doi:10.1371/journal.pone.0099581)
Supplement: Table S2 — Parameter estimates. Proportion of replicates where the true value lies within the 10%, 50% and 90% confidence intervals of the posterior distribution. , , . (PDF) [file pone.0099581.s007.pdf]

Table S2. Parameter estimates. Proportion of replicates where the true value lies within the 10%, 50% and 90% confidence intervals of the posterior distribution. $N_B = 0.1N$, $n = 20$, $l = 30$.

		$\theta = 0.0015$				$\theta = 0.005$			
		θ	T	N_B	All	θ	T	N_B	All
	10%	0.056	0.227	0.225	0.009	0.093	0.17	0.235	0.005
TPH	50%	0.298	0.839	0.839	0.27	0.486	0.797	0.931	0.432
	90%	0.797	1	1	0.797	0.884	1	1	0.884
	10%	0.058	0.115	0.144	0.002	0.578	0.197	0.263	0.074
$\mathtt{SFS}_3$	50%	0.332	0.646	0.678	0.186	0.959	0.854	0.893	0.795
	90%	0.991	0.987	0.997	0.979	1	0.999	1	0.999
	10%	0.08	0.149	0.078	0.001	0.122	0.18	0.223	0.019
$\mathtt{T+SFS}_3$	50%	0.454	0.69	0.627	0.416	0.555	0.811	0.905	0.517
	90%	0.906	0.995	0.999	0.904	0.937	0.988	1	0.934
	10%	0.099	0.126	0.129	0.003	0.11	0.112	0.132	0.004
TPH+DH	50%	0.503	0.691	0.684	0.287	0.503	0.582	0.543	0.218
	90%	0.923	0.978	0.994	0.904	0.892	0.967	0.955	0.847
